# The reliability, validity and responsiveness of the Dutch version of the Oxford elbow score

**DOI:** 10.1186/1749-799X-6-39

**Published:** 2011-07-30

**Authors:** Jeroen de Haan, Harold Goei, Niels WL Schep, Wim E Tuinebreijer, Peter Patka, Dennis den Hartog

**Affiliations:** 1 Department of Surgery-Traumatology, Westfriesgasthuis, P.O. Box 600, 1620 AR Hoorn, The Netherlands; 2 Department of Surgery-Traumatology, Erasmus MC, University Medical Center Rotterdam, P.O. Box 2040, 3000 CA Rotterdam, The Netherlands

## Abstract

**Background:**

The Oxford elbow score (OES) is an English questionnaire that measures the patients' subjective experience of elbow surgery. The OES comprises three domains: elbow function, pain, and social-psychological effects. This questionnaire can be completed by the patient and used as an outcome measure after elbow surgery. The aim of this study was to develop and evaluate the Dutch version of the translated OES for reliability, validity and responsiveness with respect to patients after elbow trauma and surgery.

**Methods:**

The 12 items of the English-language OES were translated into Dutch and then back-translated; the back-translated questionnaire was then compared to the original English version. The OES Dutch version was completed by 69 patients (group A), 60 of whom had an elbow luxation, four an elbow fracture and five an epicondylitis. *Quick*DASH, the visual analogue pain scale (VAS) and the Mayo Elbow Performance Index (MEPI) were also completed to examine the convergent validity of the OES in group A. To calculate the test-retest reliability and responsiveness of the OES, this questionnaire was completed three times by 43 different patients (group B). An average of 52 days elapsed between therapy and the administration of the third OES (SD = 24.1).

**Results:**

The Cronbach's α coefficients for the function, pain and social-psychological domains were 0.90, 0.87 and 0.90, respectively. The intra-class correlation coefficients for the domains were 0.87 for function, 0.89 for pain and 0.87 for social-psychological. The standardised response means for the domains were 0.69, 0.46 and 0.60, respectively, and the minimal detectable changes were 27.6, 21.7 and 24.0, respectively. The convergent validity for the function, pain and social-psychological domains, which were measured as the Spearman's correlation of the OES domains with the MEPI, were 0.68, 0.77 and 0.77, respectively. The Spearman's correlations of the OES domains with *Quick*DASH were -0.43, -0.44 and -0.47, respectively, and the Spearman's correlations with the VAS were -0.33, -0.38 and -0.42, respectively.

**Conclusions:**

The Dutch OES is a reliable and valid 12-item questionnaire that can be completed within several minutes by patients with elbow injuries. This Dutch questionnaire was useful as an outcome measure in patients with elbow trauma.

## Introduction

Patient-reported outcome measures (PROMS) quantify the patients' or populations' subjective experience in relation to a health condition and its therapy [1]. It is important to measure quality of life for several reasons [2]. A patient's self-assessment of their own quality of life may differ from the judgement of the medical staff, especially with symptoms such as pain. PROMS can reveal this difference of judgement in routine clinical practice. In addition, PROMS can be used in research studies to compare two different treatments. Quality of life measures can be categorised as generic or specific for diseases or conditions [1]. The Oxford elbow score (OES) is a specific questionnaire that measures the quality of life of patients with disorders of the elbow joint [3]. The OES was designed to measure the outcomes of elbow surgery from the patient's perspective. The OES is a 12-item, patient-reported questionnaire, which makes it an important outcome measure that is independent of the evaluation of the medical team. In the Netherlands, the *Quick*DASH questionnaire (Disability of the Arm, Shoulder and Hand Questionnaire) is used to measure the state of the upper extremities before and after therapy [4]. The 11-item *Quick*DASH questionnaire is a shortened version of the 30-item DASH questionnaire, which was designed to measure physical function and symptoms in patients with musculoskeletal disorders of the upper limbs. Both DASH and *Quick*DASH have two four-item optional modules, one related to performing sports and/or playing a musical instrument and one related to work. The test-retest reliability of *Quick*DASH in a study of 101 patients was 0.90 [5]. The DASH questionnaire has been examined for reliability and validity in a group of 50 Dutch patients [6], and in that study, the Cronbach's alpha coefficient was 0.95, and the test-retest reliability, calculated as the Pearson's correlation, was 0.98, although this is not a test of agreement. This questionnaire, however, was not specifically developed to assess the elbow region [7]. The *Quick*DASH questionnaire also differs from the OES because it only asks patients about their experiences during the preceding week, whereas the OES asks patients about the preceding four weeks. The OES includes three domains: an elbow function domain, a pain domain (severity and time of day when the pain occurs) and a social-psychological condition domain; each domain is assessed using four questions. The answers are recorded on a five-point Likert scale. Every domain score is calculated to a final score that ranges from 0 (worst) to 100 (best) [3]. In a study of 104 patients who had undergone a combined total of 107 elbow operations for osteoarthritis, rheumatoid arthritis, post-traumatic stiffness and epicondylitis, the OES was found to be both reliable and valid [3]. In another study, this questionnaire was found to have a good responsiveness or ability to detect changes six months post-surgery [8]. The difference in the patients' scores before versus after elbow surgery was higher with the OES than with the DASH questionnaire.

The aim of the present study was to develop and evaluate the reliability, validity and responsiveness of the Dutch language version of the OES.

## Patients and Methods

The 12 items of the OES were translated into Dutch according to the generally accepted rules for translation of non-Dutch questionnaires [9-11]^1^. The OES was translated into Dutch by four clinicians involved in orthopaedic trauma surgery. One clinician was an epidemiologist with experience in clinimetrics. The four translated versions were compared, and the differences were resolved by discussion. The Dutch version of the OES was then back-translated to English by a certified English translator (and native English speaker). The four clinicians compared this back-translation with the original English version of the OES, and they edited the Dutch translation to make it more accurate. After the translation process, mistakes were encountered in the tense of the Dutch version of questions seven and eight, which referred to pain during the past four weeks. These mistakes were found after the back-translation and were corrected.

The OES was validated by calculating the Spearman's rank correlation with *Quick*DASH, the Mayo Elbow Performance Index (MEPI) [12] and the visual analogue scale for pain (VAS) [13]. The MEPI is one of the most widely used physician-rated classification systems for elbow function and its relation to the overall quality of life [14]. This index consists of four parts: pain, ulnohumeral motion, stability and the ability to perform five functional tasks [12]. The MEPI was chosen for validation because it is an objective, physician-rated questionnaire that is available in the Netherlands. The pain level was determined with a 10-point VAS, in which zero implied no pain and ten implied the worst possible pain. The VAS was chosen because it provides a simple way to record subjective estimates of pain intensity, and the fact that pain has a large influence on questionnaires that assess elbow function [15].

To validate the Dutch OES, the present study examined a cohort of 69 patients (group A) who were seen for elbow trauma at four clinical sites. Patients 15 years of age or older with a simple or complex elbow dislocation (n = 60), epicondylitis (n = 5) or fracture in the elbow region (n = 4) were included from four hospitals (three rural teaching hospitals and one university hospital). The patients with previous elbow dislocations were in a chronic stage with a mean follow-up of 3.3 years, and the other nine cases were in an acute stage. Patients younger than 15 years and patients unable to read Dutch were excluded from the study. The elbow dislocations were treated either with plaster or with a sling for two weeks. The elbow fractures were reduced and internally fixated. The patients with epicondylitis were injected locally with platelet-rich plasma. Sixty-nine patients completed the OES and *Quick*DASH, and 58 patients completed the VAS for pain.

The MEPI was completed by the physician for 49 patients, and four domains were assessed: pain (maximum score of 45 points), ulnohumeral movement (maximum score of 20 points), stability (maximum score of 10 points) and the patient's ability to accomplish five functional tasks (maximum score of 25 points). The five functional tasks were 1) the ability to comb one's hair, 2) the ability to feed oneself, 3) the ability to perform personal hygiene tasks, 4) the ability to put on a shirt and 5) the ability to put on one's shoes.

The patient's pain level was assessed with the following question, "How much pain do you have in your elbow?" This question was scored using a 10-point VAS for pain, with 0 indicating no pain and 10 indicating the worst possible pain imaginable.

*Quick*DASH is a standardised and validated questionnaire that assesses a patient's symptoms and disabilities at work and during leisure activities [4]; the *Quick*DASH questionnaire can be downloaded free of charge from the following website: http://www.dash.iwh.on.ca. This questionnaire, which assesses the entire upper extremity, was completed by the patients themselves. The *Quick*DASH questionnaire consists of three modules. The first module includes 11 questions about symptoms and the ability to perform certain activities. The second and third modules, which are both optional, contain four questions each. The first optional module asks questions about how the patient is affected at work, and the other module asks questions about how they are affected while playing sports or a musical instrument. All of the questions are scored on a five-point scale. The total score of each of the three modules is summed and corresponds to an overall score on a scale of 0 (no disabilities) to 100 (severe disabilities). All three of the modules were used for the present analysis. Lastly, the validity of the Dutch OES was measured by calculating the correlation between the Dutch OES, *Quick*DASH, the VAS for pain and the MEPI.

In a separate cohort (group B) of 43 patients, the OES was administered three times. The elbow dislocations in this second group B were either treated with plaster or with a sling for three weeks. The elbow fractures were reduced and internally fixated. After the operation patients were allowed to exercise. The patients with epicondylitis were injected locally with platelet-rich plasma. The timing of the administration of the second OES differed between patients and was performed after a median time-period of one day (interquartile range = 6.0). The second test allowed us to calculate the test-retest reliability.

The OES test was also administered a third time to the patients of group B; this third administration allowed us to analyse the ability of the OES to detect changes in patient status (i.e., to determine its responsiveness). An average of 52 days elapsed between therapy and the administration of the third OES (SD = 24.1, minimum 28 days, maximum 103 days), as clinically detectable changes were expected after the treatment of the elbow fractures and dislocations. The first administration of the OES in group B was performed during the acute stage of the disorder, with a mean of 16.6 days (SD = 22.6, minimum -7 days, maximum 86 days) after the therapy to increase the possibility of observing a change between the first administration and third administration of the OES. The OES refers to the period of "the past 4 weeks", and the interval between the trauma and the administration of the OES reduced the possibility of problems for those patients with an acute trauma to complete the questionnaire.

### Statistical Analyses

The questionnaires were imported into the PASW Statistics 18.0 software package and analysed using the same computer program. The test reliability was analysed by calculating the Cronbach's α coefficient and the intra-class correlation coefficient (ICC). As a measure of test-retest agreement for each domain, the standard error of measurement was calculated by dividing the mean difference in score between the initial test and the retest by the square root of two [16]. Using the standard error of measurement, the minimal detectable changes (MDC) of the three domains were calculated using the following formula: MDC = 1.96*√2*standard error of measurement [16]. The standard error of measurement and MDC were both expressed on the same scale of measurement as the OES (i.e., 0-100).

The convergent validity was estimated by calculating the Spearman's correlation coefficients among the OES scores and those for *Quick*DASH, the VAS for pain and the MEPI. Spearman's correlation coefficients were used because the data of the questionnaires were not normally distributed.

The ability of the OES to detect changes in patient status (i.e., responsiveness or longitudinal validity) was calculated by determining the effect size and the standardised response means. The effect size was calculated by dividing the difference in patients' scores between the first administration and third administration of the OES by the standard deviation of the score from the first administration. The mean standardised response was calculated by dividing the mean change in score by the standard deviation of the change in scores.

The percentages of scores below 25 and above 75 for the three domains of the OES were calculated to assess floor and ceiling effects.

## Results

The patient characteristics are presented in Table 1. The mean age of the patients in group A was 43.4 (SD = 14.8) years and 50.9 (SD = 12.8) years in group B. In group A, 52 of the total patients (75%) were female, whereas in group B, 27 patients (63%) were female.

**Table 1:** Patient characteristics.

Characteristics	Group A	Group B
**N**	69	43
**Gender (N)**		
**female**	52	27
**male**	17	16
**Age (years)**	43.4 (SD = 14.8)	50.9 (SD = 12.8)
**Diagnosis (N)**		
**elbow dislocation**	60	19
**elbow fracture**	4	14
**epicondylitis**	5	5
**arthrolysis**		2
**other elbow conditions**		3

The outcomes of the OES analysis are shown in Table 2. By removing the question "How would you describe the pain you usually had from your elbow?" from the pain domain, Cronbach's α coefficient of this domain increased slightly to 0.90. Removal of any other questions decreased the Cronbach's α coefficient for the respective domain. When a single question from the function domain, either question 1, 2, 3 or 4, was removed from the analysis, the Cronbach's α coefficients were 0.87, 0.87, 0.88 or 0.87, respectively. When either question 7, 8, 11 or 12 (from the pain domain) was removed from the analysis, the Cronbach's α coefficients were 0.78, 0.79, 0.86 or 0.90, respectively; the Cronbach's α coefficients were 0.88, 0.87, 0.85 or 0.89, when question 5, 6, 9 or 10 (from the social-psychological domain), respectively, was removed from the analysis.

**Table 2:** Results of the analysis of the OES.

	OES domains		
	
	Function	Pain	Social-psychological
**Mean score (SD) pre-intervention data**	66.7 (28.8)	69.2 (27.5)	62.5 (30.2)
**Cronbach's α pre-intervention data**	0.90	0.87	0.90
**Intra-class correlation coefficient (95% CI)**	0.87 (0.75, 0.93)	0.89 (0.78, 0.94)	0.87 (0.73, 0.93)
**Standard error of measurement**	9.9	7.8	8.7
**Minimal detectable change**	27.6	21.7	24.0
**Effect size**	.56	.49	.54
**Standardised response mean**	.69	.46	.60
**% scores < 25 pre-intervention data**	16.2	11.1	19.2
**% scores > 75 pre-intervention data**	42.5	47.6	38.4

CI = confidence interval

The Spearman correlation coefficients among the three domains of the OES and *Quick*DASH, the VAS for pain, and the MEPI (which were calculated to evaluate the convergent validity of the OES among the patients in group A) are shown in Table 3.

**Table 3:** Correlation between the three domains of the Oxford elbow score, the *Quick*DASH domains, the visual analogue pain scale (VAS), and the Mayo elbow performance index (MEPI).

Oxford elbow score domain	Function N = 69	Pain N = 69	*Quick*DASH total N = 69	*Quick*DASH work N = 53	*Quick*DASH sports/music N = 48	VAS pain N = 58	MEPI N = 49
**Function**			-.43**	-.23	-.35*	-.33*	.68**
**Pain**	.85**		-.44**	-.32*	-.42**	-.38**	.77**
**Social-psychological condition**	.84**	.89**	-.47**	-.38**	-.46**	-.42**	.77**

**p < 0.01 and *p < 0.05. Spearman's correlation coefficients were calculated to assess the relationship between the results of each OES domain and the questionnaires listed above.

## Discussion

In the present study, the reliability (expressed as Cronbach's α coefficient for internal consistency) and the test-retest reliability of the Dutch version of the OES were both high for all three of the domains. In a study by Dawson et al., the Cronbach's α coefficients for the three domains were also found to be high: for the elbow function domain, it was 0.90; for the pain domain, it was 0.89; and for the social-psychological domain, it was 0.84; the ICC values for each domain in this study were 0.89, 0.98 and 0.87, respectively [3].

The effect sizes and standardised response means, which are a measure of the test's responsiveness or its ability to detect changes in patients' conditions, were moderate. This finding was in contrast to the study of Dawson et al., which found that the English OES domains had large effect sizes (i.e., 0.79, 1.14 and 1.18 for the function, pain and social-psychological domains, respectively) [8]. This difference in effect sizes and standardised response means can be explained by our shorter period of follow-up at the third administration of the OES. Except for pain, the standard error of measurement and the MDC measurements of the three domains were comparable to those in the Dawson et al. study [8]. The standard error of measurements for the function, pain and social-psychological domains in the Dawson et al. study were reported to be 8.23, 3.58 and 8.51, respectively, and the MDCs were 18.73, 8.25 and 18.85, respectively [8]. The difference in the standard error of measurement and MDC for pain can be explained by the different time period between the first administration and second administration of the OES in our study (interquartile range = 6.0 days) and the study of Dawson et al. (an interval of 2 days for all of the patients) [3,8]. Terwee et al. also found a large variation in the values of minimal important change of PROMS by the same method across studies and across different methods within studies [17]. The authors stated that caution was needed when interpreting and using published minimal important change values.

The distribution of the domain scores showed that a high percentage of patients had superior scores above 75. This finding could point to a ceiling effect of the OES, which is a failure to detect differences between patients with a high score; differences at the high end of the scale could be too small to reliably distinguish individuals. But it is usual to obtain skewed scores in opposite directions for pre and postsurgical interventions in orthopaedics and ceiling effects are more relevant to item level rather than to the overall score analysis.

The correlation between the three domains of the Dutch version of the OES and the MEPI was high, which indicates that the OES has a good convergent validity. The MEPI score was mainly determined by the contribution of elbow pain (45%) to the patients' overall elbow functioning. Doornberg et al. have concluded that pain has a large influence on questionnaires that assess elbow function, both those that are completed by physicians and those that are completed by the patients [15]; however, it should be noted that Doornberg et al. did not examine the OES in their study. In our study, the correlation between the OES and the *Quick*DASH questionnaire was moderate. Dawson et al., however, have found a high degree of correlation between the 30-item DASH and the function domain of the OES (-0.84) but only a moderate degree of correlation between the DASH and the pain (-0.66) and social-psychological domains (-0.59) [3]. Interestingly, in a continuation study that was performed in a different patient population, Dawson et al. found a moderate correlation between the OES and the 30-item DASH (-0.51, -0.54 and -0.58 for the function, pain and social-psychological domains, respectively), which was more in accordance with our findings [8]. The moderate correlation between the OES and *Quick*DASH can be attributed to the difference in time recall because the *Quick*DASH questionnaire asks patients about the preceding week, and the OES addresses the past four weeks. The VAS for pain had a moderate correlation with the OES, which was probably because the OES assesses a patient's pain under specific circumstances, such as during the night. In contrast, the VAS for pain assesses a patient's mean overall pain intensity at the present moment and does not ask if the degree of pain changes under specific circumstances.

This study had several limitations, including the small sample sizes and a homogeneous patient population (i.e., patients with elbow trauma) in the two studied cohorts. The reliability of a measuring instrument in classical test theory is characteristic of the sample tested. Another limitation of this study was the variation in the time that elapsed between the first administration and second administration of the OES as well as between the first administration and third administration. The OES refers to the preceding four weeks, and, during this period, the patients were treated for their elbow dislocation with a plaster or sling, which could have interfered with their movements that were addressed by the questions of the OES. This problem could have affected the correlations with the other instruments which used different periods of recall. The variability in time between the administrations could have lowered the ICCs of the OES domains. In addition, the variability in the length of time between the OES administrations could have influenced the standard error of the measurements, the MDC and the effect size measures.

Because our patient population included a relatively homogeneous group, future studies should examine OES results in patients with other types of elbow disorders. An analysis of the OES via modern test theory would also be necessary to examine the ordering of the five scoring categories.

## Conclusion

The Dutch OES is a reliable and valid 12-item questionnaire that can be completed within several minutes by patients with elbow injuries. This Dutch questionnaire was useful as an outcome measure in patients with elbow trauma, and the Dutch language version can now be applied in the Dutch population.

Future studies will use this Dutch OES in a randomised controlled trial for the evaluation of the functional treatment of simple elbow dislocations [18]. In addition, the Dutch OES will be used in an observational study of surgeries of complex elbow dislocations.

## Specified notice

Oxford Elbow Score^© ^Isis Innovation Limited, 2008. All rights reserved.

The authors, being Professor Ray Fitzpatrick and Dr. Jill Dawson, have asserted their moral rights.

## Competing interests

The authors declare that they have no competing interests.

## Authors' contributions

JDH, NWLS, HG, WET and DDH developed the study and drafted and revised the manuscript. WET performed the statistical analysis of the data. JDH, DDH and HG participated in patient inclusion and assessment. All of the authors have read and approved the final manuscript.
